# Examining the relationship between sports participation and youth developmental outcomes for socially vulnerable youth

**DOI:** 10.1186/s12889-018-5955-y

**Published:** 2018-08-15

**Authors:** Sabina Super, Niels Hermens, Kirsten Verkooijen, Maria Koelen

**Affiliations:** 1 Chair Group Health and Society, Department of Social Sciences, Wageningen University, P.O. Box 8130, 6700 EW Wageningen, the Netherlands; 2 School of Governance, Utrecht University, Bijlhouwerstraat 6, 3511 ZC Utrecht, the Netherlands

**Keywords:** Positive youth development, Sense of coherence, Sport, Socially vulnerable youth, Self-regulation skills, Life prospects

## Abstract

**Background:**

Research has shown that sports participation is positively related to youth developmental outcomes, but it is still unknown if sports participation relates to these outcomes among socially vulnerable youth. Hence, this research aimed to examine the relationship between sports participation and youth developmental outcomes (i.e., problem behaviour, pro-social behaviour, school performance, subjective health, well-being, self-regulation skills, and sense of coherence) for socially vulnerable youth. In addition, the stability of the relationship between sports participation and the youth developmental outcomes were investigated with a six-month interval.

**Methods:**

Two identical questionnaires were administered with a six-month interval by youth professionals from four youth organisations, measuring the youth developmental outcomes and sports participation rates of socially vulnerable youth. In total, 283 socially vulnerable youths (average 14.68 years old) participated at baseline and 187 youths after six months.

**Results:**

The results showed that sports participation was positively related to pro-social behaviour, subjective health, well-being, and sense of coherence at both measurements. We found no evidence for the relationship between sports participation and problem behaviour and the self-regulatory skills. In addition, sports participation was only positively related to school performance at the first, but not at the second, measurement.

**Conclusions:**

The results of this study show that there are positive relationships between sports participation and several youth developmental outcomes. Based on the current data no conclusions can be drawn about the causal relationship between sports participation and youth developmental outcomes. Given the focus of policymakers and health professionals on sport as a means to achieve wider social and educational outcomes for young people, including in the Netherlands, further research is needed to shed light on the relationship between sports participation and youth developmental outcomes for socially vulnerable youth, with a special focus on this group’s heterogeneity.

**Trial registration:**

Trialregister.nl NTR4621 Date of Registration: 2 June 2014 (retrospectively registered).

## Background

Researchers and policymakers have often advocated that sports participation can be beneficial for the personal development of young people [1, 2] as studies have found evidence that sports participation can benefit not only physical health, but also mental, cognitive and social health (see for reviews [3, 4]). The Human Capital Model developed by Bailey [3], for example, gives a comprehensive overview of six different forms of capital that showed to have a positive relationship with sports participation: physical capital, emotional capital, individual capital, social capital, intellectual capital and financial capital. Underlying the Human Capital Model is the assumption that competencies, skills and knowledge can be acquired by participating in sport resulting in positive youth development. The evidence base for the different forms of capital gains from sports participation is diverse, with strong evidence supporting physical capital gains but with weaker evidence for individual capital gains or financial capital gains. Nonetheless, the sports setting is often considered an avenue for positive youth development [1].

Organising inclusive sports activities is considered to be especially relevant for *socially vulnerable youth* who are characterised by an accumulation of negative experiences with the institutions in their lives [5]. The negative experiences with institutions can relate to the family domain (e.g., the parents have financial problems or youths experience domestic violence), to the school domain (e.g., youths are bullied at school), to the judicial system (e.g., after drug use or after a crime) or to the community (e.g., living in a bad neighbourhood with high crime rates). These negative experiences lead to distorted and disconnected relationships with those institutions [5] and as a result socially vulnerable youth are often confronted with feelings of incompetence, rejection, isolation and a low self-esteem. Considering that socially vulnerable youth participate less often in sport than their non-vulnerable peers [6], there is great potential to engage these young people in a pedagogical and supportive setting. However, the relationship between sports participation and developmental outcomes amongst socially vulnerable youth is hardly investigated.

### The present study

A large body of evidence is available that suggests that sports participation is positively associated with more healthy behaviours [7, 8], improved school performance [9, 10], improved subjective health [3, 11], and increased well-being [12, 13] in young people. However, this research has paid little attention to investigating this relationship among *socially vulnerable youth groups*. Recent reviews examining the effects of sports programs on the personal development of *socially vulnerable youth* also concluded that very little research has been conducted among this specific youth group [14, 15] and that the effects of sports participation on youth development were inconsistent. Indeed, Ten Broeke demonstrated that knowledge in developmental psychology is largely based on research that has been conducted in Western, Caucasian, and middle-class research populations [16]. Yet, Henrich et al. [17] argue that the results from studies among these WEIRD populations – White, Educated, Industrialised, Rich, and Democratic – are the least representative to generalise to other populations. Because there is still limited and inconsistent evidence regarding the relationship between sports participation and youth development amongst socially vulnerable youth, there is a need for further research. Hence, this first aim of this study is to investigate, among socially vulnerable youth, the relation between sports participation and indicators of youth development, as measured in: (a) behaviour, (b) school performance, (c) subjective health, and (d) well-being.

The second aim of this study is to investigate, among socially vulnerable youth, the relation between sports participation and two proximal outcomes: self-regulation skills and sense of coherence. The first proximal outcome, self-regulations skills, refers to a specific set of assets that may be relevant for the longer-term developments in the distal outcomes behaviour, school-performance, subjective health, and well-being. These self-regulations skills are: planning, self-evaluation, monitoring, effort, reflection, and self-efficacy [18]. Self-regulation is considered to have an influence on a person’s success [19] in the broadest sense of the word and in various societal domains [20–22]. Self-regulatory skills have previously been found to correlate positively with young people’s sports participation [22–25]. A study by Jonker et al. [23] demonstrated that pre-university students (12–16 years) participating in sport scored higher on planning, reflection, and effort than their pre-university peers that did not participate in sport. Posner and Rothbart [26] state that the development of self-regulation in children is influenced by both genes and the environment in which children live. Specific exercises during childhood, especially attention training, can improve self-regulation skills. In this respect, it has been claimed that youths that participate in sport have increased opportunities to train and develop self-regulation skills [25]. In addition, it has been pointed out that people develop self-regulatory skills best in inspiring environments that are rich in feedback and that require goal-setting [22], characteristics that are frequently present in the sports setting. According to Piché et al. [24], there exists a mutual relation between sports participation and self-regulation. The authors found that kindergarten childhood participation in physical activity predicted self-regulation skills in the fourth grade. Moreover, they found that kindergarten childhood self-regulation skills predicted participation in physical activity in the fourth grade. Current studies on the relationship between sports participation and self-regulation focussed only to a limited extent on vulnerable youth groups.

The second proximal outcome, sense of coherence, explains people’s capacity to cope with stressful life challenges in a health-promoting way [27, 28]. Sense of coherence has a vital role in orienting a person towards understanding a specific stressor (i.e., comprehensibility), in evaluating the resources that might be available to deal with everyday life stressors (i.e., manageability), and in engaging with challenges as a meaningful process (i.e., meaningfulness). Individuals with a relatively strong sense of coherence are better able to comprehend the stressors that they encounter in everyday life and have a general confidence that resources are available to meet the demands posed by stressful situations [27]. Furthermore, they consider stressors more as a meaningful challenge than as a threat and, hence, they are better able to select effective coping mechanisms, resolving tension in a health-promoting manner. Previous studies have found a positive relationship between sports participation and sense of coherence [29–31]. Yet, to the best of the authors’ knowledge, this relationship has not been studied in vulnerable youth groups.

The third aim of this study is investigate the stability of the relationship between sports participation and youth developmental outcomes. Research has shown that socially vulnerable youth face a turbulent life characterised by challenges and stressors on a daily basis [5] which can influence their ability to participate in sport at a given moment [6]. In addition, how they report on developmental outcomes (e.g., subjective health or well-being) may fluctuate depending on the amount of stressors they are experiencing at a specific moment. To understand better how sports participation is related to youth developmental outcomes, the stability of this relationship should be accounted for. It is for this reason that data were collected among socially vulnerable youth by administering two identical questionnaires with a six-month interval.

Summarising, the following three study aims were formulated:

1. To investigate, among socially vulnerable youth, the relation between sports participation and indicators of youth development, as measured in: (a) behaviour, (b) school performance, (c) subjective health, and (d) well-being.
2. To investigate, among socially vulnerable youth, the relation between sports participation and self-regulation skills (i.e., planning, monitoring, effort, and reflection) and sense of coherence.
3. To investigate the stability of the relationship between sports participation and youth developmental outcomes.

## Methods

This study is part of the research project Youth, Care and Sport, set up to study the value of sport for socially vulnerable youth (see for a detailed description [32]). Cross-sectional data were collected with two identical questionnaires administered with a six-month interval among socially vulnerable youth.

### Study population

Data were collected via four youth organisations that work with socially vulnerable youth (between 12 and 23 years old). The participating youth organisations provide services to youths who are (temporarily) experiencing problems in their personal development, for example because they have learning or behavioural problems or because they live in settings that hinder this development (e.g., parents incapable of providing proper care). The services provided by these organisations include school social work and educational counselling services as well as more specialised (mental) healthcare. The youth organisations are funded by a complex mix of government subsidies and private funding. The participating youth organisations were a youth care organisation in a large Dutch city and three schools for special education of which two were located in a large Dutch city and one in a rural area.

The youth professionals employed at the participating organisations asked the youths, which were clients of the youth organisations, to participate in the study. This procedure resulted in a non-randomised, purposive sample of participants. At Time 1 (T_1_), data were collected on 283 youths. Nine youths completed less than half of the baseline questionnaire and were removed from the sample, leading to a sample size of 274 participants (209 boys and 65 girls). The average age of the youths was 14.68 (*SD* = 1.69). At the six-month follow-up (T_2_), 194 participants completed the questionnaire. After removing seven youths from the sample because they completed less than half of the questionnaire, the remaining 187 participants were used in the analyses (follow-up rate: 68.2%). The main reason for dropout was that the youths had left the youth organisation, for example because their treatment plan was finalised or because they dropped-out of school. The youths that dropped out at T_2_ were significantly older at T_1_ (*M* = 15.29, *SD* = 1.97) than the youths that completed the questionnaire at T_2_ (*M* = 14.41, *SD* = 1.47), *t*(267) = 4.062, *p* < .001. No other significant differences were found between the youths that did or did not complete the second questionnaire.

### Data collection

Data were collected via paper questionnaires that contained questions adapted to the language and cognitive skills of the study population. A pilot test was conducted within one unit of a youth organisation to see whether the questionnaire was understandable for the youths. The five participating youths indicated that the included questions were clear and comprehensible. However, to reduce the burden for the participants, the Motivational Climate Scale for Youth Sports [33] was removed from the questionnaire. On average, the youths needed between 15 and 20 min to fill in the questionnaire.

Due to the vulnerable nature of the study population, special attention was paid to obtaining informed consent. An information letter that contained detailed information about the aim and the set-up of the study was sent to the parents. The letter included information about the confidential use of the data for this research and guaranteed parents that the data would not be distributed to third parties, would not be discussed with the youth professionals, and would be solely used for the research project Youth, Care and Sport. Parents were asked to contact the youth professional if they objected to their child’s participation in the study (i.e., passive informed consent). The youth professionals involved in the data collection were instructed by the researchers about the data collection procedure. These instructions also included the ethical aspects of administering the questionnaires and the rights of the youths that participated in the study. Consequently, the youth professionals that administered the questionnaires made sure that the youths knew that participation was on a voluntary basis and that they had the right to stop participating at any time without any repercussions. Youths that agreed to take part in the research project (i.e., oral informed consent) received a questionnaire from the youth professional. During the data collection, a youth professional was present to answer any of the youths’ questions regarding the items in the questionnaire. The questionnaires were administered in various settings, but mostly in a classroom setting or at the youth’s home. After completion of the second questionnaire (T_2_), the youths received a gift voucher for their participation. This project was performed in accordance with the code of conduct for minors [34] and with general ethical guidelines for behavioural and social research in the Netherlands, peer-reviewed, and approved by the review board of the Wageningen School of Social Sciences.

### Measures

Demographic data were gathered regarding the participant’s age, sex, and the youth organisation responsible for collecting the data (T_1_). The following measures were included in the two questionnaires:

#### Distal youth developmental outcomes

Four distal youth developmental outcomes were included in the questionnaire: (a) behaviour, (b) school performance, (c) subjective health, and (d) well-being. In order to assess *behaviour*, the Strengths and Difficulties Questionnaire (SDQ) was administered [35]. This instrument has often been used as a screening tool for behavioural disorders [36, 37], and the psychometric properties have previously been found satisfactory in a Dutch sample of non-vulnerable children and adolescents [38]. The SDQ contains five sub-scales of five items each: hyperactivity (example item: “I am restless, I cannot stay still for long”), emotional symptoms (example item: “I worry a lot”), conduct problems (example item: “I often have temper tantrums or hot tempers”), peer problems (example item: “I have one good friend or more”), and pro-social behaviour (example item: “I am helpful if someone is hurt, upset, or feeling ill”). The items could be scored on a three-point scale: ‘not true’, ‘somewhat true’, and ‘certainly true’. Following Goodman’s [35] procedures, a total SDQ score was calculated by using the subscales hyperactivity, emotional symptoms, conduct problems, and peer problems (T_1_ α = .73; T_2_ α = .72). Higher total SDQ scores reflect a higher rate of behavioural disorder. The fifth subscale, pro-social behaviour, was computed by taking the average of the five pro-social items, and higher scores reflect more pro-social behaviour. The internal consistency of the pro-social behaviour scale was marginal (T_1_ α = .61; T_2_ α = .67). As a self-developed indicator of *school performance*, youths were asked to report how their teacher was likely to evaluate their work. The five-point scale ranged from ‘bad’ to ‘excellent’. The youths’ *subjective health* was assessed using a question from the Short Form Health Survey (SF-36) [39]. Both at T_1_ and T_2_, youths answered the following question “In general, how good is your health?” on a five-point scale ranging from ‘bad’ to ‘excellent’. Finally, the youths were asked to answer the following question “How are you currently feeling?” on a five-point scale ranging from ‘bad’ to ‘excellent’, as an indicator of *well-being*.

#### Proximal youth developmental outcomes

Two proximal youth developmental outcomes were included in the questionnaire: (a) self-regulation skills, and (b) sense of coherence. The *self-regulation skills* were assessed using the Self-Regulation of Learning Self-Report Scale [18]. The original scale consisted of six subscales, but, to reduce the burden for the participants, four subscales were selected for this study. The selection was based on previous research that indicated that participation in sport was most strongly related to these four scales [22] and on the relevance of these scales for the purpose of this study. All the items could be scored on a four-point scale ranging from ‘almost never’ to ‘almost always’. Example items were: “I determine how to solve a problem before I begin” (planning), “I check how well I am doing when I solve a task” (monitoring), “I concentrate fully when I do a task” (effort), and “I try to think about my strengths and weaknesses” (reflection). Scores on the subscale items were averaged, with higher values representing stronger self-regulatory skills. The internal consistency of the scales was satisfactory: planning (eight items, T_1_ α = .85; T_2_ α = 87), monitoring (six items, T_1_ α = .78; T_2_ α = .82), effort (nine items, T_1_ α = .83: T_1_ α = .83), and reflection (five items, T_1_ α = .80; T_2_ α = .88). *Sense of coherence* was measured using the Dutch translation of the Orientation to Life Questionnaire (SOC-13) adapted to young people [40]. The 13 items of this scale could be scored on a five-point scale from ‘almost never’ to ‘almost always’, with the exception of two items that were positively formulated and could be scored from ‘very bad’ to ‘very good’ (T_1_ α = .83; T_2_ α = .84). Example items are: “How often has it happened that people who you counted on disappointed you?” and “How often do you have feelings that you’re not sure you can keep under control?” A sum score was calculated for the 13 items, with higher scores reflecting a stronger sense of coherence.

#### Sports participation

Measurements regarding the youths’ sports participation were based on the Dutch Guideline for Sport Participation Research (Richtlijn Sportdeelname Onderzoek (RSO)), with recall periods adapted to fit the timeframe of this research [41]. The questions were preceded by a short explanation of the definition of sports participation, to make sure that all participants understood what sports participation entailed: “Examples of sport are football, badminton, fitness, and bike tours, but not doing puzzles, walking a dog, or cycling to school. Physical activity during school times (physical education or playing outside) is not included”. The items included in the questionnaire addressed the (a) frequency of sports participation in the previous month, (b) frequency of sports participation on average per week (c) average duration of sports activity, (d) the type of sports played, and (e) membership of a sports or fitness club. The variable *frequency of sports participation in the previous month* was an open-ended question. Strong doubts were raised by the youth professionals about the reliability of the variable *frequency of sports participation in the previous month* as the youths were often unable to correctly answer this question. This observation led to the decision to drop this variable from the analysis. The variable *frequency of sports participation on average per week* had five answer categories: ‘once a week’, ‘2 times a week’, ‘3 times a week’, ‘4 times a week’, and ‘5 or more times a week’. The variable *average duration of sports activity* had five answer categories: ‘less than half an hour’, ‘between an half and 1 hour’, ‘between 1 and 2 hours’, ‘between 2 and 3 hours’, and ‘longer than 3 hours’.

### Data analysis

All statistical analyses were carried out using IBM SPSS version 23. The internal consistency of the variables was obtained using Cronbach’s alpha. Mean and standard deviations were inspected, as well as the distribution properties of the variables. The following continues variables were not approximately normally distributed: total SDQ score, pro-social behaviour, effort, and reflection. The data for total SDQ score, pro-social behaviour, and effort were transformed using the square root function, after which the variables were approximately normally distributed. The reflection scale remained not normally distributed and was dropped from the analysis since no reliable outcomes would be obtained from a statistical test. To see whether there were differences between the youths across the four youth organisations, the T_1_ variables were compared across the participating youth organisations using ANOVA for the normally distributed variables and using Kruskal-Wallis for the ordinal variables school performance, subjective health and well-being. A paired-samples t-test was conducted to see if the average scores differed between T_1_ and T_2_ for the continues variables and the Wilcoxon signed-rank test for the ordinal variables.

To examine the relationship between sports participation and the *total SDQ* score and *pro-social behaviour,* the *self-regulation skills*, planning, monitoring and effort, and *sense of coherence*, we used a repeated measures analysis of variance, where the participants’ age and sex were included as covariates (ANCOVA). The between-subjects factor (i.e., Group factor) in the analysis was based on the variable *frequency of sports participation on average per week* at T_2_*.* In order to have relatively equal group sizes, participants were divided in three groups of sports participation: no-sport group, moderate-sport group (1 or 2 times a week), high-sport group (3 or more times a week). For all variables, all assumptions for conducting repeated measures ANCOVA were met: no outliers were detected, there was homogeneity of variance (as assessed by Levene’s test), and homogeneity of covariances (as assessed by Box’s test). Eta squared is reported for all the continues variables as a measure of effect size.

For the ordinal variables, *school performance*, *subjective health*, and *well-being*, a Mantel-Haenszel test of trend was run to determine whether a linear association existed between the variables and the frequency of sports participation (i.e., the three groups of sports participation). The three groups of sports participation at T_1_ served as the between-subjects factor in the analysis for the T_1_ variables and the three groups of sports participation at T_2_ served as the between-subjects factor for the T_2_ variables. Following the analysis of main group differences, for the ordinal variables *school performance*, *subjective health*, and *well-being,* we calculated a change score indicating a negative development (− 1), no change (0), or a positive development (1). We used the Mantel-Haenszel test of trend to see whether the change scores differed across the three groups of sports participation at T_2_.

## Results

Table 1 shows the participants’ characteristics at T_1_ and T_2_. Seventy percent of the youths participated in a sport in the previous month at T_1_ and at T_2_. At T_1_, the most popular sports were soccer, fitness, swimming, and boxing. Of the 187 youths that completed both questionnaires, 37 youths did not participate in a sport (19.9%), 15 youths started to participate in a sport (8.1%), 20 youths stopped participating in a sport (10.8%) and 114 youths continued participating in a sport (61.3%). Sixty-seven percent of the youths remained in the same sports-group (i.e., no-sport, moderate-sport, and high-sport) between T_1_ and T_2_. Of the youths that participated in a sport at T_2_, 42.7% played a sport under supervision of a sports coach or a sports leader. No significant differences were found for the T_1_ variables between the four youth organisations. In addition, the paired-samples t-test showed that the average scores on the outcome variables did not differ between T_1_ and T_2_ (*p* > .29).

**Table 1:** Characteristics of participants at Time 1 and Time 2 (*N* = 187) and *p*-values for the outcome variables

Variable	Time 1 (T_1_)				Time 2 (T_2_)				*p*-value
	N	%	M	SD	N	%	M	SD	
Personal characteristics									
Sex	187								
Boys	149	79.7							
Girls	38	20.3							
Age (years)	186		14.41	1.47					
Youth development									
SDQ (total)	170		10.89	5.00	171		11.18	4.88	.67
Pro-social behaviour	185		7.42	1.90	184		7.36	1.89	.55
School performance	187		3.30	0.82	187		3.29	0.83	.84
Subjective health	187		3.44	1.05	187		3.43	0.92	.76
Well-being	187		3.40	1.00	187		3.35	0.86	.59
Assets									
Self-regulation skills									
Planning	147		2.42	0.58	141		2.46	0.62	.71
Monitoring	149		2.54	0.57	139		2.50	0.64	.48
Effort	149		2.69	0.51	137		2.67	0.51	.34
Sense of coherence									
Sense of coherence	178		34.09	7.82	180		33.52	7.77	.29
Sports participation									
Frequency of sport (average week)	186				186				
Did not sport	50	26.9			54	29.0			
1 or 2 times a week	70	37.6			63	33.8			
3 or more times a week	66	35.5			69	37.1			
Duration of sport (average per activity)	185				185				
Did not sport	50	27.0			55	29.7			
< ½ an hour	7	3.8			9	4.9			
Between ½ - 1 h	17	9.2			13	7.0			
Between 1 and 2 h	93	50.3			95	51.4			
Between 2 and 3 h	10	5.4			9	4.9			
Longer than 3 h	8	4.3			4	2.2			
Membership of sports/fitness club	186				185				
No	84	45.2			97	52.4			
Yes	102	54.8			88	47.6			

*SDQ total* Strengths and Difficulties. Scale range: SDQ total (0–40); pro-social behaviour (0–10); school performance, subjective health, and well-being (1–5); planning, monitoring, and effort (1–4); reflection (1–5); sense of coherence (0–52)

The repeated measures ANCOVAs yielded a significant main group effect for pro-social behaviour and sense of coherence (see Table 2). A similar trend was observed for the total SDQ score. Post-hoc analysis with Bonferroni correction revealed that, for pro-social behaviour, the high-sport group scored significantly higher than the no-sport group (*p* = .004). For sense of coherence, the moderate-sport group scored significantly higher than the no-sport group (*p* = .001). No significant difference was found for sense of coherence with the high-sport group (*p* = .139). The repeated measures ANCOVA yielded non-significant main effects for Time (*p* > .170) and a non-significant Group x Time interaction effect (*p* > .198) for all the variables. There was a main effect of sex for pro-social behaviour, *F*(1, 175) = 4.713, *p* = .031, ɳ^2^ = .026, and effort, *F* = (1, 129) = 4.490, *p* = .036, ɳ^2^ = .034, where girls scored higher than boys on both pro-social behaviour and effort. In addition, there was a main effect of age for planning, *F* (1, 128) = 6.036, *p* = .015, ɳ^2^ = .045, and monitoring *F* (1, 127) = 7.522, *p* = .007, ɳ^2^ = .056, where older youths scored higher on both self-regulatory skills.

**Table 2:** Means, standard deviations, and main effects for group on the outcome measures (*N* = 180)

Group	Time 1						Time 2								
	no sport		moderate		high		no sport		moderate		high				
Variable	M	SD	M	SD	M	SD	M	SD	M	SD	M	SD	F	p	ɳ^2^
Problem behaviour	12.31	5.25	10.18	4.63	10.47	5.02	12.20	4.90	10.68	4.70	10.95	4.91	2842	.061	.036
Pro-social behaviour	6.92	1.99	7.51	1.79	7.68	1.88	6.87	1.65	7.25	1.87	7.82	1.99	4307	.015	.047
Planning	2.50	0.59	2.31	0.50	2.49	0.64	2.41	0.61	2.32	0.63	2.57	0.61	1374	.257	.021
Monitoring	2.54	0.52	2.44	0.51	2.59	0.65	2.40	0.60	2.47	0.61	2.56	0.66	0.450	.638	.007
Effort	2.59	0.50	2.67	0.45	2.78	0.56	2.58	0.53	2.66	0.51	2.75	0.49	1909	.152	.029
Sense of coherence	31.11	8.02	36.30	7.27	34.53	7.71	31.24	8.06	35.81	7.24	33.33	7.72	6374	.002	.072

*M* mean, *SD* standard deviation, *F* statistics obtained from the between-subjects effects; the grouping variable is “average frequency of sports participation per week” at T_2_: no-sport group (0), moderate-sport group (once or twice a week), high-sport group (three or more times a week); the internal consistency of the variables: Problem behaviour (T_1_ α = .73; T_2_ α = .72), pro-social behaviour (T_1_ α = .61; T_2_ α = .67), planning T_1_ α = .85; T_2_ α = 87), monitoring (T_1_ α = .78; T_2_ α = .82), effort (T_1_ α = .83: T_1_ α = .83), sense of coherence (T_1_ α = .83; T_2_ α = .84)

For the ordinal variables (i.e., school performance, subjective health, and well-being) at T_1_, the Mantel-Haenszel test of trend showed a statistically significant linear association between the groups of sports participation and school performance χ^2^(1) = 9.054, *p* = .003, *r* = .22, subjective health χ^2^(1) = 12.988, *p* < .001, *r* = .27 and, well-being χ^2^(1) = 12.340, *p* < .001, *r* = .26. Higher frequency of sports participation was associated with higher scores on school performance, subjective health, and well-being. At T_2_, the Mantel-Haenszel test of trend showed a statistically significant linear association between the groups of sports participation and subjective health χ^2^(1) = 15.649, *p* < .001, *r* = .29 and well-being χ^2^(1) = 6.145, *p* = .013, *r* = .18, but not with school performance χ^2^(1) = 0.365, *p* = .546, *r* = .04. Higher frequency of sports participation was associated with higher scores on subjective health and well-being.

The Mantel-Haenszel test of trend showed a statistically significant linear association between the groups of sports participation at T_2_ and the *change score* of school performance χ^2^(1) = 5.316, *p* = .021, *r* = .17. There were no significant associations between the groups of sports participation at T_2_ and the *change scores* of subjective health and well-being.

## Discussion

The aim of this article was to examine the relationship between sports participation and youth development outcomes in a Dutch socially vulnerable youth group. Moreover, we examined the stability of this relationship within a 6-month interval. We found that 70% of the socially vulnerable youth participated in sport at least once a week in the month prior to the questionnaire, at both measurements. In addition, almost two thirds of the youths kept on playing a sport in the six months between the two questionnaires. We found a positive relationship between sports participation and pro-social behaviour, subjective health, well-being, and sense of coherence. These findings proved to be stable across the two measurements. We found no evidence for the relationship between sports participation and total SDQ score (i.e., problem behaviour) and the self-regulatory skills. In addition, sports participation was only positively related to school performance at the first, but not at the second, measurement.

Contrary to our expectations [24], we found no evidence for the positive relationship between sports participation and the self-regulatory skills planning, monitoring and effort. An explanation for the absence of a positive relationship between sports participation and the self-regulatory skills can be grounded in the discussion whether self-regulatory skills are domain-general skills or domain-specific skills. Several authors have suggested that self-regulatory skills are domain-general skills that are relevant for several performance domains [22]. In other words, self-regulatory skills such as planning and effort can be used in various life domains interchangeably, such as in the sports setting or in the school setting. However, other researchers have found contradicting results suggesting that metacognitive skills, such as the self-regulatory skills, are domain-specific [42]. This means that young people may report high scores on the self-regulatory skills planning and effort within the sports setting, but at the same time report low scores on these skills in other life domains. The Self-Regulation of Learning Self-Report Scale, included in this study, measured domain-general skills. As the questionnaires were mostly administered in classroom settings, it is possible that youths reflected on their skills in relation to their school performance. This may explain why we did not find a relationship with sports participation. More research is needed to understand the relationship between sports participation and self-regulatory skills among socially vulnerable youth.

In this current study we found that sports participation was positively related to sense of coherence. Sense of coherence reflects a person’s ability to cope with stressful events in a health-promoting way [27, 28]. As socially vulnerable youth are confronted with stressors on a daily basis, a stronger sense of coherence may be an important factor in determining the youths ability to deal with these stressors and, subsequently, increasing the chance that they are able to participate in sport. The other way around, the sports setting may be a setting in which socially vulnerable youth have life experiences that are known to be conducive to the strengthening of sense of coherence: consistency, load-balance, and socially-valued decision making. García-Moya et al. [43] examined the contextual factors contributing to the development of sense of coherence in children aged 13 to 18 years. The most important predictor of sense of coherence was the quality of parent–child relationships, but other contexts (i.e., the school, the neighbourhood, and peer relations) also remained important in predicting sense of coherence. Consequently, the authors [43] concluded that “*contextual factors seemed to predominantly act in an additive fashion*” (p. 919) suggesting that the sports setting could aid in strengthening the sense of coherence next to other important life domains. Further research on the development of sense of coherence, specifically within the sports setting, may be especially interesting because sense of coherence reflects a life orientation that can be used throughout the life-course, in different settings and situations [28, 44]. People with a strong sense of coherence are better able to use the resources they have available to deal with everyday life challenges. Therefore, the influence of the availability of assets (e.g., self-regulation skills) on individuals’ healthy development may depend on the level of sense of coherence. It would, therefore, be interesting to investigate whether young people with a relatively strong sense of coherence are better able than young people with a relatively weak sense of coherence to transfer life skills from the sports setting to other life domains.

The findings in this study partially corroborate existing evidence on the positive relationship between sports participation and youth developmental outcomes (see for an overview for example: [3, 4, 45]). It is important to note that research has pointed out that reciprocal relationships exist between sports participation and the outcomes that were measured in this study. For example, it has been demonstrated that behavioural problems can be a barrier to sports participation [46, 47], suggesting that behavioural problems predict sports participation rates, as well as the other way around. Similarly, in a large German cohort study, Manz et al. [46] found that having psychopathological problems (measured with the Strengths and Difficulties Questionnaire) was a predictor of abstaining from organised sports participation. It was also found that having emotional symptoms correlated with lower levels of physical activity in a cohort study with 10-year-old children [47]. These findings support the idea that youths’ developmental status may also determine the chance that they participate in sport. It is within this context, that researchers call for inclusive sports activities as a first step in reaching positive youth development, recognising that the youths’ developmental status is also influential in the youths’ potential to participate in sport [48].

Sports participation is not a unified concept as it can take many shapes and forms. Coalter [49] makes a distinction between *sport* activities, *sport-plus* activities, and *plus-sport* activities. *Sport* activities include both recreational and competitive sport, where the focus lies on playing a sport in the hope that this will lead to changes in youth developmental outcomes [49, 50]. *Sport-plus* activities also focus on sport, but within these activities sport is seen as an important setting for positively influencing youth developmental outcomes. Additional non-sport components are added to the activities that aim to facilitate this change process. For example, each training can be organised around a specific life skill in which certain exercises are included to train the particular life skill. Finally, *plus-sport* activities focus mainly on youth development and use sport as a vehicle to attract young people and to positively influence youth developmental outcomes. Sport in these *plus-sport* activities is often broadly defined (e.g., game playing). For youths who are more ‘at risk’, it has been suggested that sports activities should shift more towards *plus-sport* activities in order to achieve positive outcomes [50]. The current study focused on the Dutch sports sector, which is organised around national sports federations with members going to local sports clubs. These sports clubs are often run by volunteer sports coaches who receive only limited or no formal coaching training and, hence, there is very little or no attention of the pedagogical aspects of the sports setting [51]. Acknowledging that intentionally structuring and designing the sports setting to reach positive youth development is important [52], it is not surprising that we did not observe a change in the different youth development outcomes (except for school performance) across time.

Research has suggested that for the positive development of socially vulnerable youth it is perhaps not the frequency or duration of their sports participation that is of importance, but rather the exposure to a supportive, motivational and pedagogical climate [53, 54]. A mastery motivational climate, which focuses on personal effort, improvement and mastery, has been positively linked to enjoyment [55] and the motivation to continue participating in sport. [56]. Studies have also supported the observation that the motivational climate is an important predictor of the reported youth developmental outcomes [57–59]. Even more so, a negative or unbalanced sports climate could harm individual players and potentially push youths further down the spiral of vulnerability [60, 61]. To fully understand the relationship between sports participation and youth development, assessing the quality of the sports climate, and thus the quality of the developmental experiences for youths [62], is necessary.

### Strengths and limitations

This study is, to the best of the authors’ knowledge, unique in investigating the association between sports participation and youth developmental outcomes for socially vulnerable youth. First of all, 283 young people participated in the first round of the questionnaire thanks to a strong network of youth organisations involved in the project Youth, Care and Sport. This made it possible to assess the association between sports participation and various indicators of youth development for a large group of vulnerable young people. Secondly, this is the first study to assess the outcomes at two time points, allowing us to examine the stability of the association between sports participation and youth developmental outcomes. And finally, whereas previous studies have often focused on specific sports-based interventions or programs, this study has focused on the traditional sports sector that is dominant in many Western European countries. In this respect, this study has contributed to a number of insights into this rapidly developing area of research.

A recent review of the social and emotional well-being of at-risk youth participating in physical activity programs showed that the risk of bias was high in all the included studies, for example because very few studies included a control group or effect sizes [15]. This current study was unable to overcome these biases. The original study, as described in the study protocol [32], had a non-equivalent control group design with an intervention implemented in the experimental condition that aimed to increase the sports participation of socially vulnerable youth. However, due to the changing context in which our research project was conducted, it was no longer possible to implement the intervention. The most important change concerned a political transition in the organisation of the Dutch youth care system during which the responsibility of organising youth care shifted from the youth care organisation with whom we worked to the local government. This transition did not only delay the start of the data collection, but also required the researchers to seek collaboration with a new party (i.e., the local government) that was now responsible for deciding on the content of the youth professionals’ work. This ultimately led to the abolishment of the intervention and the connected non-equivalent control group design. The researchers also encountered several challenges such as building trust with the youth professionals, obtaining parental consent, and attrition rates. The challenges that researchers experience when conducting research in vulnerable groups often disrupt research or prevent it from being conducted [63]. We have tried to deal with these challenges throughout the project in the best possible way in an attempt to gain valuable data of an under-researched population. Nonetheless, a number of limitations have to be borne in mind concerning the results presented in this paper.

First of all, due to changes in the original study design, this current study did not have an intervention group and a control group. The absence of a (quasi-)experimental design prevents us from drawing conclusions about causal relationships. Following Webb’s [64] recommendations for further research in the positive youth development area, longitudinal and prospective designs are needed to assess developmental changes through sports participation and “*to analytically separate them from the influences of other social and structural factors on youth development*” (p. 178).

Secondly, we divided participants into three groups based on the average number of times per week they participated in sport. Future research may benefit from a more accurate and precise measurement of sports participation, by also including the intensity of the sports activity. Furthermore, the sample size of this study did not allow us to investigate whether youths that started or stopped participating in sport differed on developmental outcomes from youths that kept on participating or did not participate in sport between the two questionnaires. Future research could investigate how youth developmental outcomes may differ across sports participation patterns, using longitudinal designs.

A third limitation that needs to be considered is the heterogeneity of the sample. All the participants faced, temporarily or over a longer period of time, problems in growing up. However, the degree to which the participants were socially vulnerable might have differed to a large extent. The youth organisations involved in this study offer services to youths with a wide range of problems such as being bullied in school, having autism or ADHD, having parents with drug or alcohol problems, and so forth. For ethical reasons, we were unable to collect any information about the problems that the youths were facing. Yet, the extent to which people experience being socially vulnerable is very relevant for how they experience their participation in sport [65]. More detailed information about the youths’ problems would have allowed us to investigate whether sports participation could have different outcomes for different groups of vulnerable youths. The lack of these insights makes it unrealistic to make generalisations about the positive associations between sports participation and the youth developmental outcomes for socially vulnerable youth. Moreover, a large proportion of this study’s participants were boys. Although boys are over-represented in the Dutch youth care system [66] – in 2015, 58.5% of all youths receiving youth care were boys – this does limit our ability to generalise the findings of the current study to all socially vulnerable youth and to socially vulnerable girls specifically.

This study did not take into account other extracurricular activities in which the youths may have been involved in addition or alternatively to their participation in sport. A study by Larson et al. [67] demonstrated that different organised activities have a very distinct profile of developmental experiences. Community-oriented activities, for example, scored high on developmental experiences related to adult networks and social capital. Similarly, performance and fine arts activities scored high on developmental experiences related to initiative. Future research could include a broad set of extracurricular activities to see how sports participation and other extracurricular activities relate to a healthy development among socially vulnerable youth.

## Conclusion

This study investigated the relationship between sports participation and youth developmental outcomes in a Dutch socially vulnerable youth population and examined the stability of this relationship with a 6-month interval. We found a positive relationship between sports participation and pro-social behaviour, subjective health, well-being, and sense of coherence. These findings were stable across the two measurements. We found no evidence for the relationship between sports participation and total SDQ score (i.e., problem behaviour) and self-regulatory skills. In addition, sports participation was only positively related to school performance at the first, but not at the second, measurement. Based on the current data no conclusions can be drawn about the causal relationship between sports participation and youth developmental outcomes. Given the focus of policymakers and health professionals on sport as a means to achieve wider social and educational outcomes for young people, including in the Netherlands, further research is needed to shed light on the relationship between sports participation and youth developmental outcomes for socially vulnerable youth. Future research needs to focus specifically on the heterogeneity of the socially vulnerable youth group and the role of a motivational sport climate in achieving positive development outcomes.

## Abbreviation

SDQ: Strengths and Difficulties Questionnaire
